# Rheumatoid arthritis patient antibodies highly recognize IL-2 in the immune response pathway involving IRF5 and EBV antigens

**DOI:** 10.1038/s41598-018-19957-z

**Published:** 2018-01-29

**Authors:** Marco Bo, Magdalena Niegowska, Gian Luca Erre, Marco Piras, Maria Giovanna Longu, Pierangela Manchia, Mario Manca, Giuseppe Passiu, Leonardo A. Sechi

**Affiliations:** 1 Department of Biomedical Sciences, Section of Microbiology and Virology, University of Sassari, Viale San Pietro 43b, 07100 Sassari, Italy; 2 UOC Reumatologia, Dipartimento di Medicina Clinica e Sperimentale, Azienda-Ospedaliero Universitaria di Sassari, Sassari, Italy; 3 Centro Trasfusionale, ASL Sassari, Sassari, Italy

## Abstract

Rheumatoid arthritis (RA) is a chronic autoimmune disease characterized by a progressive joint damage due to largely unknown environmental factors acting in concert with risk alleles conferring genetic susceptibility. A major role has been attributed to viral infections that include past contacts with Epstein-Barr virus (EBV) and, more recently, to non-protein coding sequences of human endogenous retrovirus K (HERV-K) integrated in the human genome. Molecular mimicry between viral and self proteins is supposed to cause the loss of immune tolerance in predisposed hosts. There are evidences that anti-IL-2 antibodies (Abs) are present in subjects affected by autoimmune diseases and may be responsible for alterations in regulatory T cell responses. In this study, we evaluated the levels of Abs against IL-2, viral epitopes and interferon regulatory factor 5 (IRF5) in 140 RA patients and 137 healthy controls (HCs). Ab reactivity reached the highest levels for IRF5, EBV and IL-2 (56%, 44% and 39%, respectively) in RA with significantly lower values among HCs (7–9%, *p* < 0.0001), which suggests a possible cross-reaction between IRF5/EBV homologous antigens and shifts in T cell balance disrupted by anti-IL-2 Abs.

## Introduction

Rheumatoid arthritis (RA) is a chronic autoimmune disease of complex pathogenesis that leads to a progressive disability and major systemic complications with resulting socioeconomic concerns and premature death. Common manifestations involve synovial tissue inflammation and hyperplasia, autoantibody production, cartilage and bone destruction along with systemic features including pulmonary and cardiovascular complications^1^. RA is caused by largely unknown environmental factors acting in concert with risk alleles conferring genetic susceptibility^2^; among the former ones, a major role is attributed to viral infections putatively associated with numerous autoimmune diseases. The contribution of past contacts with Epstein-Barr virus (EBV) to RA pathogenesis has been postulated for over 30 years^3^. Although EBV resides stably at low levels during the lifetime in about 95% of the adult population worldwide^4^, molecular mimicry between viral and self proteins is supposed to cause the loss of immune tolerance in predisposed hosts^5–9^. More recently, human endogenous retrovirus K (HERV-K) has been called into question^10–14^. Remnants of previous HERV-K infections are present in the human genome as non-protein coding sequences integrated into germline cells^15^. Transcription of these elements may be activated or stimulated by exogenous viruses such as EBV resulting in the production of antigenic peptides^16^.

We have previously reported increased humoral responses to EBV and HERV-K peptides in Sardinian RA patients^17,18^ that mirrored elevated antibody (Abs) titers directed against an epitope derived from *Mycobacterium avium* subsp. *paratuberculosis* (MAP) homologous to EBV inner tegument protein BOLF1 and human interferon regulatory factor 5 (IRF5)^19^. IRF5 is known to mediate virus-induced immune responses including expression of proinflammatory cytokines and its pro-apoptotic effect is activated by EBV in transformed cells^20,21^. In systemic lupus erythematosus (SLE), IRF5 was found to negatively regulate the expression of interleukin-2 (IL-2)^22^. IL-2 is crucial for function, expansion and survival of regulatory T cells (T_reg_) and balance within this pathway is disrupted in Th1-mediated autoimmune diseases such as RA, SLE or type 1 diabetes (T1D)^23–25^. Recently, the loss of self-tolerance to IL-2 has been described in T1D subjects whose peripheral blood mononuclear cells yielded high quantities of INF-γ upon stimulation with IL-2-derived peptides^26^. Similarly, RA patients displayed raised levels of anti-IL-2 Abs supposed to affect IL-2 bioavailability necessary for T_reg_ homeostasis.

In the present study, we evaluated humoral responses to synthetic IL-2 peptides in a larger cohort of Sardinian RA patients. A correlation analysis with seroreactivity to HERV-K and homologous EBV, MAP and IRF5 epitopes permitted to assess a possible cross-reactivity of the antigens supposedly involved in RA pathogenesis. Human autoantigens along with EBV elicited the highest responses, while the strongest correlation was found between IL-2 and HERV-K pointing at a potential pathway that links EBV-induced transactivation of retroviral proteins and the subsequent cytokine secretion mediated by IRF5.

## Results

**Figure 1:**
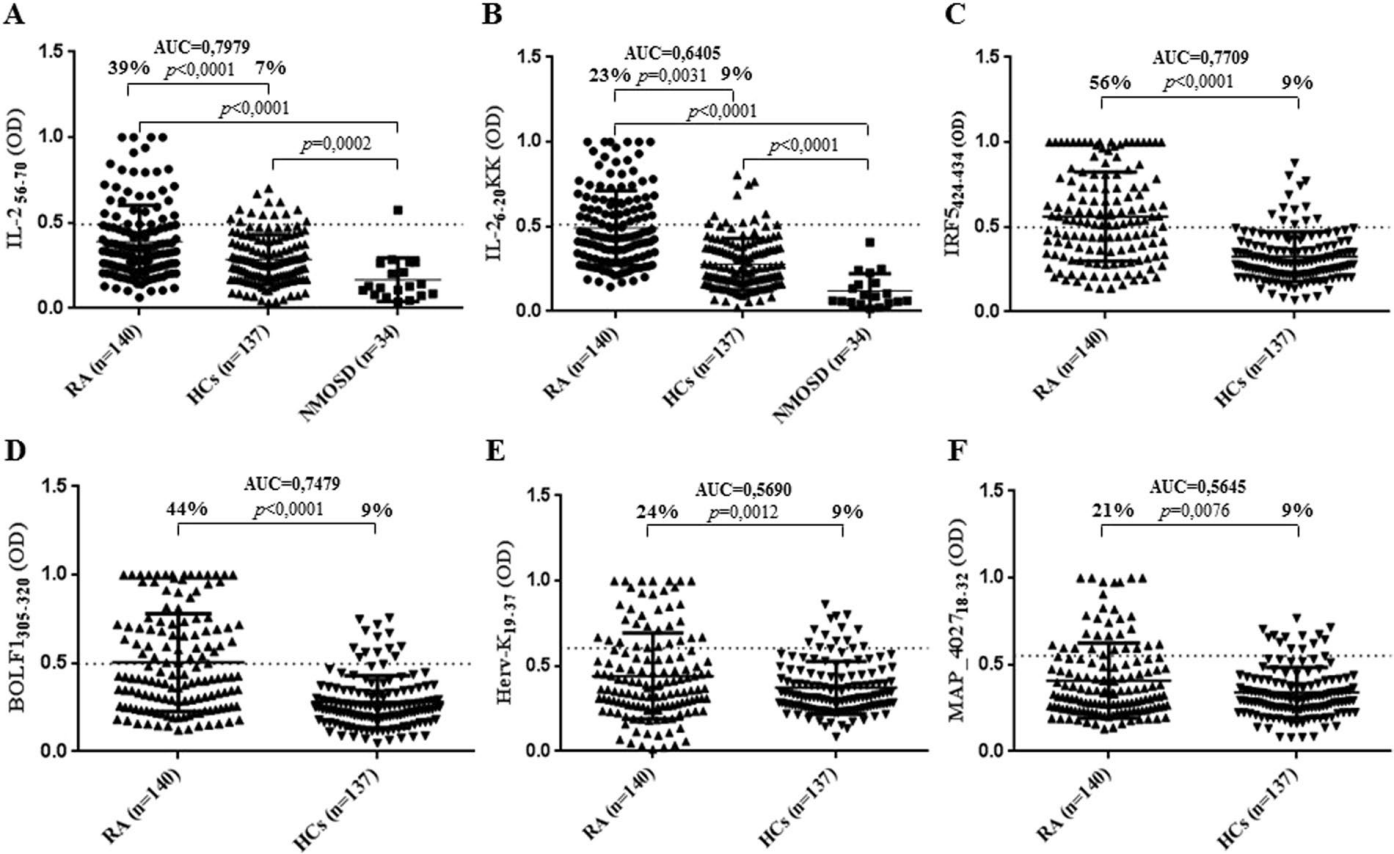
ELISA-based analysis of Abs reactivity against human, viral and MAP-derived peptides in RA, NMOSD and HCs. The sera were tested against plate-coated IL-2_6–20KK_ (**B**), IL-2_56–70_ (**A**), IRF5_424–434_ (**C**), BOLF1_305–320_ (**D**), Herv-Kenv_19–37_ (**E**) and MAP_4027_18–32_ (**F**) peptides. Bars represent the median ± interquartile range. Thresholds for Abs positivity are indicated by dashed lines. Percentages of Abs prevalence respective to each group, AUC and *P*-values are indicated above the distributions.

The potential to raise Ab responses differed between the two analyzed Il-2 peptides. IL-2_6–20KK_ elicited a higher Ab seroreactivity accounting for 39% (n = 55) among RA patients and 7% (n = 10) in HCs (p < 0.0001, Fig. 1A), while Abs against IL-2_56–70_ were detected in 23% (n = 32) of RA subjects and 8% (n = 13) of HCs (*p* = 0.0031, Fig. 1B). However, the highest levels of autoreactive Abs were directed against IRF5_424–434_ observed in 56% (n = 79) of RA patients and only 9% (n = 13) of HCs (*p* < 0.0001, Fig. 1C). Slightly lower prevalence was observed for anti-BOLF1_305–320_ Abs found in 44% (n = 61) of RA subjects and 9% of HCs (*p* < 0.0001, Fig. 1D). Responses against Herv-Kenv_19–37_ and MAP_4027_18–32_ were maintained at the same levels (9%) among HCs, while seroreactivity of RA patients equaled 24% (n = 34, *p* = 0.0012, Fig. 1E) and 21% (n = 30, *p* = 0.0076, Fig. 1F), respectively. Despite antigen-related differences in single-type Abs prevalence, all results attained statistical significance with the highest AUC values for IL-2_6–20KK_ and IRF5_424–434_.

The prevalence of Abs against both IL-2 epitopes was additionally assessed in 34 samples of patients affected by neuromyelitis optica spectrum disorder (NMOSD). Only one patients displayed values above the established cut-off for IL-2_56–70_ (2.9%, Fig. 1A) whereas lower means obtained for IL-2_6–20KK_ were mirrored by the absence of positive cases (Fig. 1B). Moreover, the responses of NMOSD patients were markedly lower compared not only to RA subjects, but also to HCs. The immunone response against the other peptides of this study in NMOSD patients has been evaluated in another study (manuscript submitted).

**Figure 2:**
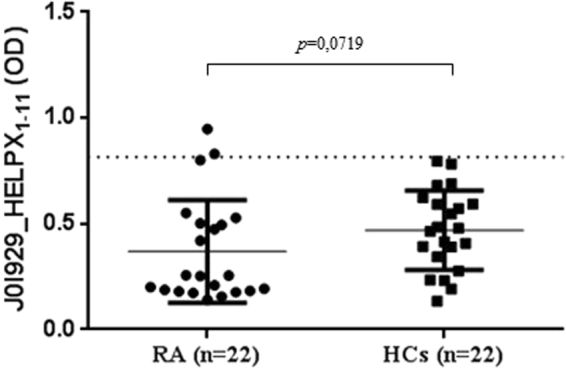
Abs reactivity against the antigenic peptide derived from *H*. *pylori* in RA patients and HCs. Bars represent mean value ± interquartile range, while dashed lines indicate the positivity threshold. Despite sequence homology to human ZnT8 protein fragment, no significant differences in Abs levels were detected.

To test the specificity of humoral responses mounted against the selected peptides, 22 HCs and 22 RA patients were randomly selected from the study population and tested for seroreactivity against J0I929_HELPX_1–11_ control peptide derived from *Helicobacter pylori* homologous to human ZnT8^27^. In both groups, half number of samples tested positive to at least one (HCs) or all (RA) of the previously assessed peptides. The observed mean values were slighty higher for HCs and corresponded to the absence of positive subjects compared to 9% (n = 2) among RA patients, however statistical significance was not attained (*p* = 0.07). Interestingly, RA individuals with multiple Abs positivity presented lower mean values compared to HCs with single-peptide positivity (Fig. 2).

**Figure 3:**
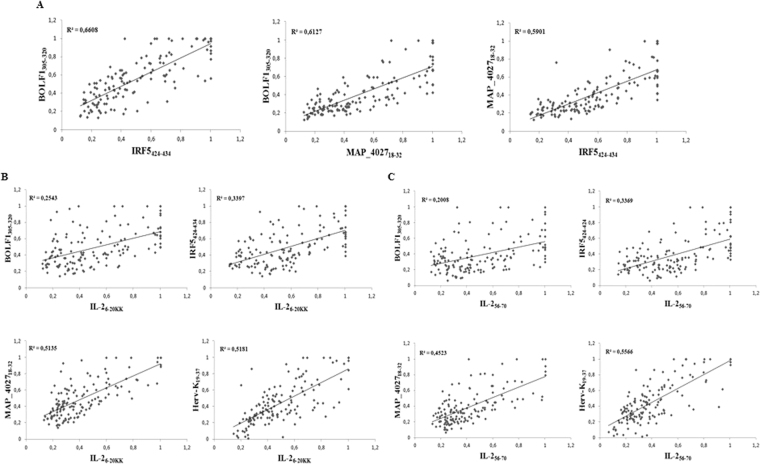
Scatter plots showing correlations between Abs titers in RA patients. Pairwise distributions are classified for homologous peptides (**A**), IL-2_6–20KK_ (**B**) and IL-2_56–70_ (**C**). Each dot correspond to OD values obtained for a single patient.

To define associations between the antigenicity of the assessed peptides we performed correlation analyses of Abs positivity values among RA patients (Fig. 3). The highest coefficients were obtained for the homologous epitopes BOLF1_305–320_, MAP_4027_18–32_ and IRF5_424–434_ in pairwise plots (Fig. 3A) pointing at cross-reactivity due to shared amino acid sequence. Correlation trends of both IL-2 peptides were similar with respect to the other antigens (Fig. 3B,C): Herv-Kenv_19–37_ and MAP_4027_18–32_ correlated moderately with either IL-2_6–20KK_ or IL-2_56–70_, however IL-2_56–70_/Herv-Kenv_19–37_ distribution corresponded to a slightly higher R^2^ value (Fig. 3B). Unexpectedly, weak to modest correlations were found between IL-2, IRF5_424–434_ and BOLF1_305–320_ (Fig. 3C).

We further evaluated multiple positivity to the assessed peptides in order to verify whether correlations between Ab titers find correspondence with the overlap in seroreactivity against IL-2 (Table 1). In contrast to mild correlations of both IL-2 epitopes plotted against IRF5_424–434_ or BOLF1_305–320_, double or triple humoral responses to these antigens were detected in a major number of subjects with distinctly higher percentages for IL-2/IRF5 reflecting single-peptide Abs prevalence. On the other hand, responsiveness to Herv-Kenv_19–37_ overlapped well with the presence of anti-IL-2_56–70_ Abs as expected from the correlation analysis and stood out also in triple positivity with positivity to IRF5_424–434_. Even though multiple responses among HCs are low in general, they visibly tend to diminish for Abs against at least three antigens.

**Table 1:** Multiple Abs prevalence in RA patients and HCs. Seroreactivity against IL-2 antigens was compared with humoral responses to MAP, EBV, HERV-K and human IRF5 peptides.

	IL-2_56–70_		IL-2_6–20 KK_						
	HCs	RA	HCs	RA	IL-2	BOLF1_305–320_	IRF5_424–434_	MAP_4027_18–32_	Herv-K_19–37_
^b^	5 (3.65)	24 (17.14)	5 (3.65)	36 (25.17)					
	2 (1.46)	27 (19.28)	4 (2.92)	43 (30.71)					
^b^	3 (2.19)	20 (14.28)	4 (2.92)	28 (20.00)					
^a^	5 (3.65)	25 (17.86)	0	29 (20.71)					
	2 (1.46)	20 (14.28)	0	24 (17.14)					
	1 (0.73)	20 (14.28)	2 (1.46)	27 (19.28)					
	1(0.73)	24 (17.14)	3 (2.19)	33 (23.57)					
	0	19 (13.57)	2 (1.46)	27 (19.28)					
	1 (0.73)	17 (12.14)	0	19 (13.57)					
	1 (0.73)	23 (16.43)	0	27 (19.28)					
	0	19 (13.57)	1 (0.73)	26 (18.57)					
	0	19 (13.57)	0	23 (16.43)					
	1 (0.73)	17 (12.14)	0	20 (14.28)					
	0	19 (13.57)	0	19 (13.57)					
	0	16 (11.43)	0	19 (13.57)					

The numbers of subjects positive for anti-IL-2_6–20KK_ and/or anti-IL-2_56–70_ Abs are reported with relative percentages in brackets. Horizontal bars indicate Abs against at least two antigens identified in the samples with IL-2 referred to as both IL-2_6–20KK_ and IL-2_56–70_. p < 0.0001 for all values except ^a^IL-2_56–70_ (p < 0.0002) and ^b^IL-2_56–70_ (p < 0.0003).

Upon sex-related screening of RA samples, females showed higher mean Abs values and positivity prevalence for all peptides compared to males, however statistical significance was reached only for IRF5_424–434_ (*p* = 0.034). After classification of RA patients and HCs in three age groups (≤49, 50–59 and ≥60), the highest responsiveness was observed for BOLF1, IRF5 and IL-2_6–20KK_ (Table 2). This trend was clearly visible in the youngest RA group regardless of sex, however females maintained it more stably until the age of 59. In contrast to men, humoral responses of RA and HC women were not significant in the oldest group but this could be affected by a small number of elderly HCs in our study population. A general decrease in Ab positivity proportional to age was common to either male or female patients. Importantly, seroreactivity to IRF5 exceeded 93% in the youngest females and reached a 100% in ≤49 year-old males (one patient).

**Table 2:** Age- and sex-related Abs prevalence in RA patients and HCs.

Gender	Age (y)	N	Subjects	BOLF1	*P*	IRF5	*P*	MAP_4027	*P*	IL-2 _**56–70**_	*P*	IL-2 _**6–20KK**_	*P*	Herv-K_**19–37**_	*P*
Females	≤49	16	RA	12 (75%)	**<0**,**0001**	15 (93,75%)	**<0**,**0001**	5 (31,25%)	**0**,**0063**	5 (31,25%)	0,088	8 (50%)	**<0**,**0001**	5 (31,25%)	0,38
		65	HCs	8 (12,30%)		6 (9,2%)		5 (7,6%)		9 (13,8%)		6 (9,2%)		9 (13,8%)	
	50–59	41	RA	21 (51,21%)	**<0**,**0001**	23 (56,09%)	**<0**,**0001**	8 (19,51%)	0,51	10 (24,39%)	**0**,**045**	20 (48,78%)	**<0**,**0001**	9 (21,95%)	0,13
		19	HCs	2 (10,52%)		1 (5,2%)		3 (15,78%)		1 (5,2%)		1 (5,2%)		1 (5,2%)	
	≥60	49	RA	17 (34,69%)	0,17	25 (51,02%)	**0**,**024**	11 (22,44%)	0,92	12 (22,44%)	0,61	17 (34,69%)	0,081	15 (30,61%)	0,17
		6	HCs	1 (16,66%)		1 (16,66%)		0		1 (16,66%)		1 (16,66%)		0	
Males	≤49	6	RA	4 (66,66%)	**0**,**0046**	6 (100%)	**0**,**0001**	3 (50%)	0,094	3 (50%)	0,302	4 (66,66%)	**0**,**0449**	2 (33,33%)	0,34
		15	HCs	2 (13,33%)		1 (6,6%)		4 (13,3%)		1 (6,6%)		2 (13,33%)		1 (6,6%)	
	50–59	11	RA	2 (18,18%)	**0**,**0023**	3 (27,27%)	0,535	1 (9%)	0,43	2 (9%)	**0**,**015**	1 (18,18%)	**0**,**0006**	2 (18,18%)	0,198
		23	HCs	0		3 (13,04%)		1 (4,3%)		1 (4,3%)		0		1 (4,3%)	
	≥60	17	RA	5 (29,41%)	**0**,**0035**	7 (41,17%)	0,104	3 (17,64%)	**0**,**0246**	1 (5,8%)	**0**,**0211**	4 (23,52%)	**0**,**0005**	1 (5,8%)	0,652
		9	HCs	0		1 (11,1%)		0		0		0		1 (11,1%)	

The numbers of individuals responsive to single antigens are provided with relative percentages. Statistically significant values are highlighted in bold.

PCA analysis permitted to identify relationships between clinical variables and the selected epitopes with 79.17% of cumulative variation describing four principal components (Table 3). Correlation between Ab values and two inflammatory parameters was low but attained a statistical significance that differed based on the analyzed measure: ESR correlated to Ab values towards IL-2 and HERV-K, while CRP yielded higher coefficients in plots with the homologous MAP, EBV and IRF5 antigens. No correlation with other clinical data was found.

**Table 3:** Correlation coefficients between inflammation measures, demographic data and seroreactivity relative to the selected antigens. All correlations are expressed as squared cosines of the variables.

	PC1	PC2	PC3	PC4
BOLF1	**0**,**629**	0,017	0,063	0,003
IRF5	**0**,**732**	0,014	0,037	0,004
MAP_4027	**0**,**820**	0,000	0,000	0,005
IL-2_56–70_	**0**,**685**	0,042	0,012	0,008
IL-2_6–20KK_	**0**,**726**	0,047	0,028	0,001
Herv-K	**0**,**596**	0,099	0,037	0,000
ESR	0,044	0,281	**0**,**475**	0,007
CRP	0,023	**0**,**660**	0,002	0,120
Age	0,041	0,065	**0**,**590**	0,015
Sex	0,054	0,206	0,004	**0**,**727**

## Discussion

Recent reports on the loss of self-tolerance to IL-2 in autoimmune diseases encouraged us to evaluate the presence of anti-IL-2 Abs in Sardinian RA patients in association to antigens most frequently described as possible contributors to RA progression. Our results confirm the involvement of IL-2 in RA at higher rates compared to a French cohort (39% vs.15%, respectively)^26^ and is mirrored by a concomitant positivity to peptide antigens derived from EBV, HERV-K, MAP or human IRF5. The latter has been linked to acute inflammation as a factor promoting polarization of macrophages towards an inflammatory phenotype in antigen-induced RA mouse models and driving Th1/Th17 responses^28–30^.

In the present study, IRF5, together with the EBV surface tegument protein BOLF1 and IL-2, triggered the greatest response even though devoid of a good correlation with IL-2. This suggests that the association between the two human autoantigens may not be proportionally dependent on Ab titers but favour autoimmunity when a tolerance threshold is surmounted. In contrast, IRF5 correlated well with homologous BOLF1 and MAP epitopes pointing at molecular mimicry that leads to a probable cross-reactivity with the assessed environmental agents to which humans are constantly exposed. This was recently confirmed by the competitive inhibition assay in our previous study^31^. For IL-2, the best correlation was obtained in the plot with HERV-K. While reactivation of endogenous retroviral protein expression may elicit serological and cell-mediated responses, an uncontrolled expansion of T_reg_ cells in subjects who lost self-tolerance to IL-2 or IRF5 may explain the development of autoimmunity. Interestingly, over 30% of our RA cohort displayed anti-IL-2/IRF5 Abs in a highly significant double positivity (*p* < 0.0001) and a more frequent multiple seroreactivity was observed in RA patients compared to healthy controls (Table 1).

Major prevalence of Abs directed against all single peptides and higher mean Abs values obtained for RA females in a sex-related analysis point at a more grave disease course proper to women and highlight the involvement of IRF5. This was mirrored by a strikingly high prevalence of Abs against IRF5 in the ≤49 year-old group independently of patients’ sex. An elevated general seroreactivity observed in the youngest group that decreases with age points at strong immune responses accompanying early disease onset.

A significant correlation between levels of anti-IL-2 Abs and measures of systemic inflammation (Table 3) is supportive of the hypothesis that anti-IL-2-driven impairment of T_reg_ activity may alter autoimmune processes and inflammatory burden. Other than expected, we did not find significant correlations between disease severity, immunosuppressive treatment, RF and ACPA status with levels and positivity of anti-IL-2 Abs. It should be acknowledged that all patients were under different immunosuppressive drugs at the moment of sample collection. The heterogeneity of treatment across subjects may have biased interpretation and significance of associations between humoral responses and inflammatory measures. Further analysis of IL-2 levels, quantification of INF-γ upon stimulation with the analyzed peptides and T_reg_ activity are needed to complete our observations. More numerous groups of the youngest patients at RA onset and elderly HCs would additionally permit to associate the efficacy of therapy in modulating serological and cell responses.

## Materials and Methods

### Subjects

Blood samples of 140 RA patients (34 males, 106 females; median age 58.95) and 137 healthy controls (HCs; 47 males, 90 females; median age 46.30) were collected in Vacutainer tubes for the separation of serum and further screening for Abs against IL-2, IRF5, MAP_4027, BOLF1 and HERV-K by indirect enzyme-linked immunosorbent assay (ELISA). RA patients who met the criteria of the American College of Rheumatology^32,33^ were enrolled from the outpatient clinic of the Rheumatology Unit, Department of Clinical and Experimental Medicine, University Hospital of Sassari, Italy. Clinical data collected during control medical visits included information relative to the duration of RA, therapy (steroids, Tocilizumab, Rituximab, Abatacept, DMARDs, Etanercept, Adalimumab, Golimumab, Infliximab and Certolizumab anti-TNF-α), levels of C-reactive protein (CRP), erythrocyte sedimentation rate (ESR) levels, positivity to rheumatoid factor and anti-cyclic citrullinated peptide (anti-CCP), Disease Activity Score-28 (DAS-28) and grade of disability defined through the health assessment questionnaire (HAQ). 34 NMOSD patients (5 males, 29 females; median age 51.32) were enrolled at the Neurology Clinic of the University Hospital of Sassari and at the Department of Neurosciences, Biomedicine and Motion, University of Verona, Italy. HCs were recruited at the Blood Transfusion Center of Sassari, Italy. Demographic, clinical and laboratory characteristics of the participants are summarized in Table 4. Ethical clearance for the study protocols was achieved from the local health authority (Azienda Ospedaliero-Universitaria, protocols 1134/L-16/04/2013 and 1192/L-04/02/2014) and all methods were performed in accordance with national and regional regulations. All participants were resident in Sardinia and signed written informed consent.

**Table 4:** Demographics, clinical history and laboratory data of RA patients and HCs.

	RA n = 140	HCs n = 137	p value
Age, yrs	59 ± 10	46 (13)	<0.001
Female sex, n (%)	106 (79.3)	90 (65.7)	0.02
Early disease, n (%)	10 (7.2)		
ACPA positivity, %	74.1		
RF positivity	74.4		
HAQ (0–3)	0.83 (0.73)		
DAS-28	3.59 ± 1.33		
CRP, mg/dL	0.8 ± 1.1		
ESR, mm/h	29 ± 24		
Steroids therapy, %	41		
DMARDs therapy, %	63.3		
Anti-TNF therapy, %	25.9		
Tocilizumab therapy, %	12.9		
Abatacept therapy, %	4.3		

Data are expressed as median ± 1 standard deviation. RA duration <12 months is reported as early disease. ACPA: anti-cyclic citrullinated peptide antibodies. RF: rheumatoid factor. HAQ: health assessment questionnaire. DAS-28: Disease Activity Score-28. CRP: C-reactive protein. ESR: erythrocyte sedimentation rate. DMARDs: disease modifying anti-rheumatic drugs. Anti-TNF: anti-tumor necrosis factor alpha.

### Antigens

The following peptides synthesized commercially at >90% purity (LifeTein, South Plainfield, USA) were included in the study: IL-2_6–20KK_ (KK-LLSCIALSLALVTNS-KK) and IL-2_56–70_ (LTEMLTFKFYMPKKA) based on Pérol *et al*. with modifications^26^, IRF5_424–434_ (VVPV–AARL-LLE), MAP_4027_18–32_ (AVVPVLAYAAARL-LL), BOLF1305_–320_ (AAVPVLAFDAARLRLLE) and Herv-Kenv_19–37_ (VWVPGPTDDRCPAKPEEEG). In addition, J0I929_HELPX_1–11_ (MIIGGGVSGCA) derived from *H*. *pylori* quinone oxidoreductase, homologous to human ZnT8^27^ was used as a control peptide. Moreover, wells containing no peptides adsorbed were included as negative control.

### Enzyme-linked immunosorbent assays (ELISA) and statistical analysis

Indirect ELISA to detect specific Abs against the selected antigens was performed as described previously^34^. The optical density (OD) was read at a wavelength of 405 nm using SpectraMax Plus 384 microplate reader (Molecular Devices, Sunnyvale, CA 94089, USA). For data normalization, a highly responsive serum with Ab reactivity fixed at 1.0 arbitrary unit (AU)/ml was included in all experiments. The results were expressed as a mean of three separate experiments and the statistical analyses were performed using Graphpad Prism 6.0 software (GraphPad Software Inc., La Jolla, CA 92037, USA). Upon determination of sample distribution through D’Agostino-Pearson normality test, values between RA patients and HCs were compared using a two-tailed Mann-Whitney *U* test with *p* < 0.05 considered statistically significant. The cut-off for positivity was established in the interval 0.49–0.60 (AU)/ml based on the receiver operating characteristic (ROC) curve (Fig. 1) with ≥90% specificity and 95% confidence interval. Fisher’s exact test was employed to compare the percentages of positive subjects in the two groups. Correlations between variables were analyzed through principal component analysis (PCA) using XLSTAT software ver. 17 (Addinsoft, New York).
